# Successful chemotherapy management of disseminated intravascular coagulation presenting with metastatic clear cell renal carcinoma: a case report and review of the literature

**DOI:** 10.1186/s13256-020-02369-x

**Published:** 2020-04-21

**Authors:** Huy Le Trinh, Vuong Thi Nguyen, Ngan Kim Mai, Bach Trung Tran, Quynh Nga Pham

**Affiliations:** Department of Oncology, Hanoi Medical University Hospital, Hanoi, Vietnam

**Keywords:** Disseminated intravascular coagulation, Clear cell renal cell carcinoma, Chemotherapy

## Abstract

**Background:**

Disseminated intravascular coagulation is a critical complication of advanced clear cell renal cell carcinoma, despite the rarity of the occurrence of disseminated intravascular coagulation in such tumors. The diagnosis of cancer-related disseminated intravascular coagulation is mostly based on clinical bleeding and laboratory test; available data suggest that treating the primary cancer also treats the disseminated intravascular coagulation. Among three reported cases of renal cell carcinoma-related disseminated intravascular coagulation in the literature, this is the first patient whose disseminated intravascular coagulation was successfully treated, in particular, with chemotherapy without any anti-disseminated intravascular coagulation therapies.

**Case presentation:**

This case is a 66-year-old Vietnamese man who presented disseminated intravascular coagulation 2 weeks after his admission for severe back pain. At admission, his initial laboratory work-up revealed only a mild thrombocytopenia with a platelet count of 93 × 10^9^/L (normal range, 150–450 × 10^9^/L) without clinical bleeding. His past medical history and family history were unremarkable. An open-biopsy was performed and the definitive diagnosis was bone metastatic clear cell renal cell carcinoma based on immunohistochemistry. Two weeks after admission, the diagnosis of disseminated intravascular coagulation was confirmed according to the International Society on Thrombosis and Haemostasis. Immediately, he was treated with a paclitaxel plus carboplatin regimen and disseminated intravascular coagulation completely disappeared after one cycle of systemic chemotherapy. Until recently, 11 months subsequent to the diagnosis of disseminated intravascular coagulation, he had been being undergoing maintenance therapy for metastatic clear cell renal cell carcinoma.

**Conclusions:**

First, an early detection of overt disseminated intravascular coagulation is essential, although disseminated intravascular coagulation in cancer presents as a chronic or even subclinical process with unique thrombocytopenia. Second, making a decision of systemic chemotherapy without delay at the time of disseminated intravascular coagulation diagnosis is the key to successful cancer-related disseminated intravascular coagulation treatment.

## Background

Clear cell renal carcinoma (CCRCC) is the most common histologic pattern of renal cell carcinomas (RCCs), accounting for approximately 75 to 85% of such tumors [1]. RCCs have a silent natural history for the multistep development of tumors; therefore, most patients are diagnosed with advanced disease. Disseminated intravascular coagulation (DIC) is a very rare presenting syndrome in solid tumors, particularly in metastatic RCCs, which is characterized by thrombosis, bleeding, or both and signs of activation of clotting and fibrinolytic system in a laboratory [2]. Only two cases of RCCs with DIC have been reported previously in the literature and the two cases died due to DIC [3, 4].

Based on current data, chemotherapy has no role in the management of advanced CCRCC due to the theory of resistance to cytotoxic agents of these cells and the development of immunotherapy and molecularly targeted therapy. Until recently, no data of methods in the treatment of DIC in CCRCC have been reported. Thus, we report a case of metastatic CCRCC presenting with chronic DIC, which was successfully managed with initial chemotherapy without any anti-DIC therapies.

## Case presentation

**Fig. 1:**
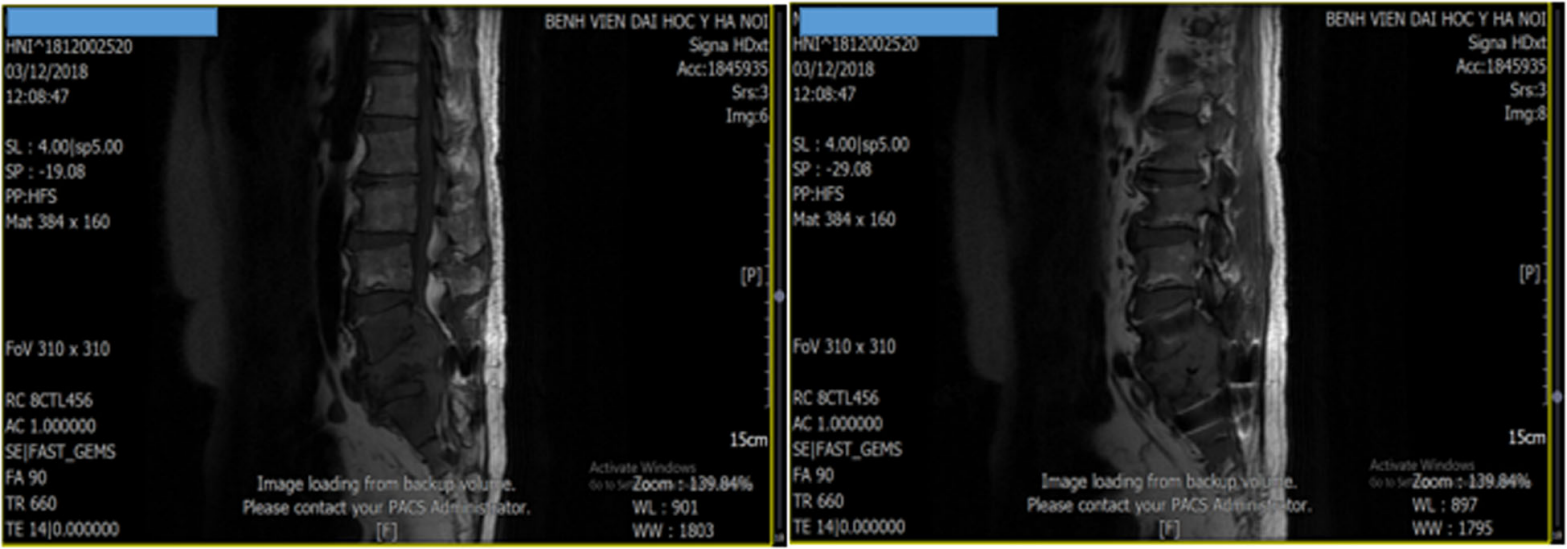
Lumbosacral spine magnetic resonance imaging revealed enlarged lytic lesions of S1 with ilium, soft tissue invasion, and compression of the nerve roots from L5 to S2 (December 2018)

This is a case of a 66-year-old Vietnamese man who was admitted to the Oncology Department at Hanoi Medical University Hospital on 3 December 2018 for severe lower back pain. He had no past medical history or family history. On examination, an initial lumbosacral spine magnetic resonance imaging (MRI) revealed enlarged lytic lesions of sacral segment 1 with ilium, soft tissue invasion, and compression of the nerve roots from L5 to S2 (Fig. 1).

**Fig. 2:**
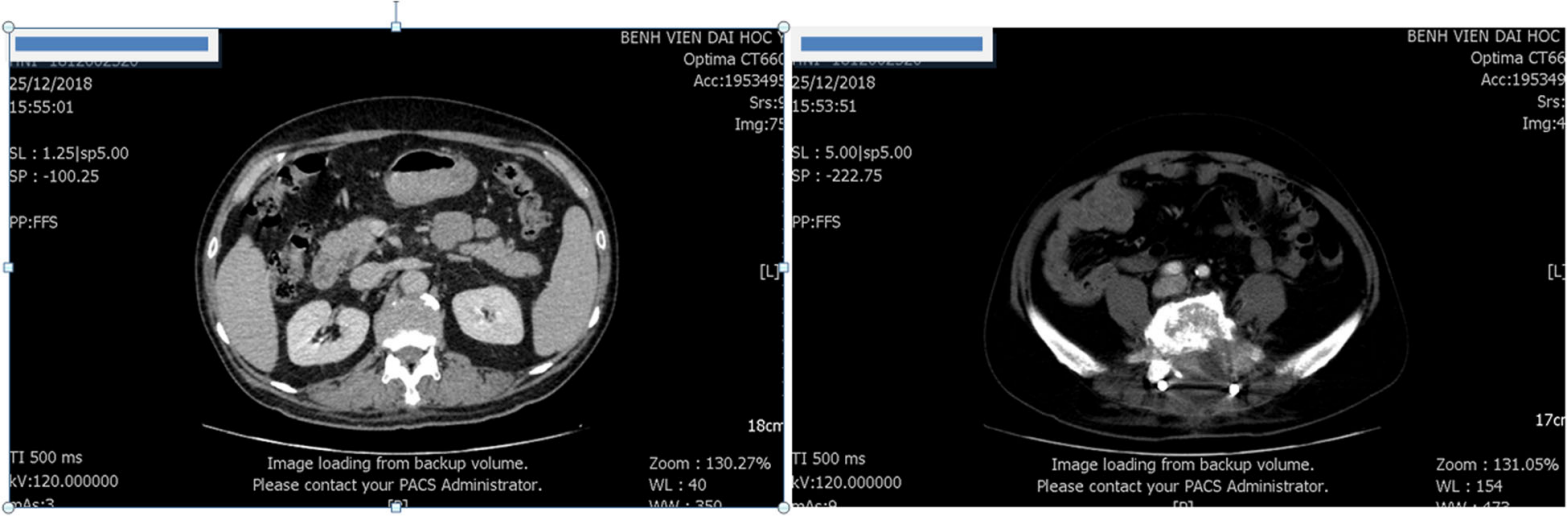
An abdominal computed tomography scan revealed enlarged lytic lesions of S1 with ilium, soft tissue invasion, and compression of the nerve roots from L5 to S2; it did not detect tumor in bilateral kidney (25 December 2018)

**Fig. 3:**
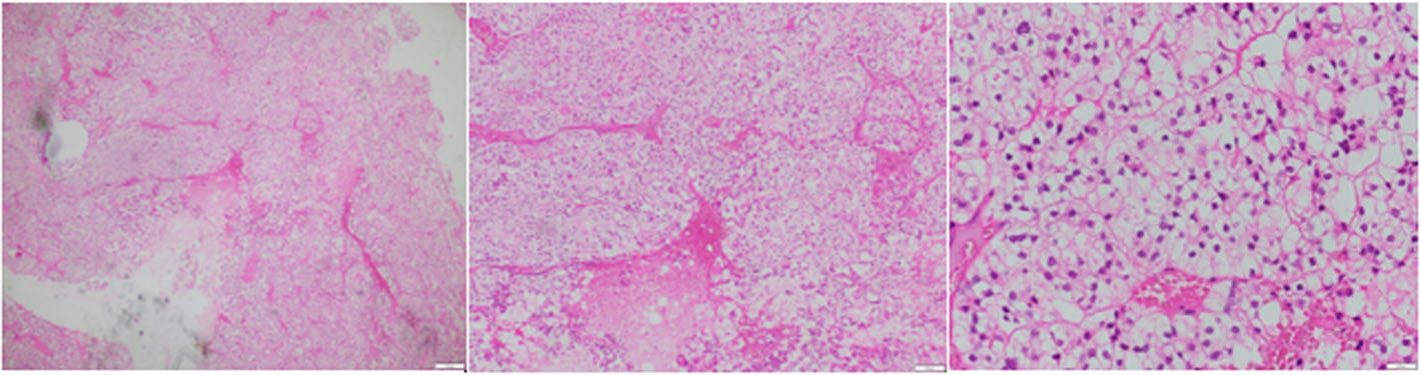
Hematoxylin-eosin staining of tumor

**Fig. 4:**
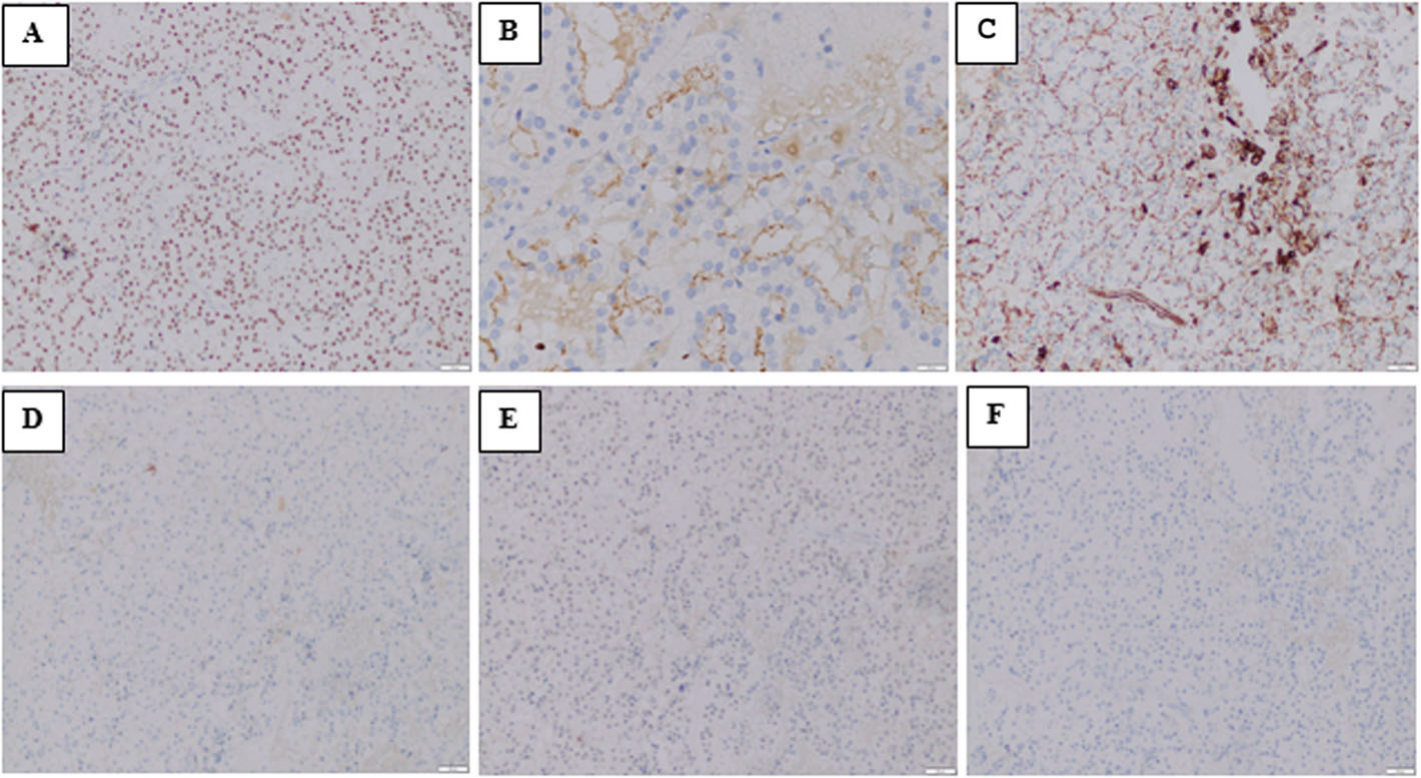
Immunohistochemistry results of tumor: **a** PAX8 positive; **b** RCC positive; **c** vimentin positive; **d** CD10 negative; **e** S100 negative; **f** chromogranin negative

At admission, initial laboratory results exhibited only a mild thrombocytopenia with a platelet count of 93 × 10^9^/L (normal range, 150–450 × 10^9^/L); his liver and kidney function were normal. Subsequent chest and abdominal computed tomography (CT) images showed no mass or abnormalities except a lytic lesion (Fig. 2). On the ninth day of admission, a surgical decompression was performed but the tumor could not be totally removed. Pathology results suggested two distinct diseases including CCRCC and parathyroid carcinoma metastasis (Fig. 3). Therefore, an immunohistochemistry (IHC) test was undertaken to acquire a confirmed diagnosis. During a period of days, while waiting for the IHC results, 2 weeks after admission, our patient complained of moderate fatigue and his laboratory data indicated thrombocytopenia with a platelet count of 78 × 10^9^/L (from 93 × 10^9^/L at baseline) and anemia with a severe hemoglobin level of 69 g/L (from 142 g/L at baseline). The calculated DIC score by the International Society on Thrombosis and Haemostasis (ISTH) was 4 [2], the non-overt DIC with D-dimer level strongly increased to 4.94 μg/mL (normal range, < 0.5 μg/mL), fibrinogen level was 2.16 g/L, and prolonged prothrombin time was 1.5 seconds. Therefore, a bone marrow biopsy was performed to rule out bone marrow involvement and the result was negative. Four days later, after 20 days of admission, our patient presented with moderate subcutaneous hemorrhage and platelet count of 51 × 10^9^/L. According to ISTH criteria, the diagnosis of DIC was confirmed with score of 5. The laboratory results associated with DIC are summarized in Table 1. Following the definitive diagnosis of DIC, he received immediate initial chemotherapy despite lack of the IHC results. Paclitaxel (175 mg/m^2^ over 3 hours) plus carboplatin (AUC = 6) regimen was indicated and repeated every 3 weeks without a transfusion of platelets or other anti-DIC therapies. One day following the first course of treatment, IHC results exhibited that tumor cells were positive for PAX, RCC, and vimentin and negative for CD10, S100, and chromogranin (Fig. 4), which supported the diagnosis of bone metastatic CCRCC.

**Table 1:** Laboratory findings associated with disseminated intravascular coagulation

	Platelet count (10^9^/l)	D-dimer (μg/mL)	Prolonged PT (seconds)	Fibrinogen level (g/l)	DIC Score
**At admission (D0)**	93 (1p)	Unmeasured	1.6 (0p)	3.18 (0p)	
**At operation (D9)**	91 (1p)	1.83 (2p)	2.7 (0p)	2.49 (0p)	3
**D15**	78 (1p)	4.94 (3p)	1.5 (0p)	2.16 (0p)	4
**D20**	51 (1p)	4.48 (3p)	3.1 (1p)	3.23 (0p)	5

*D* day, *DIC* disseminated intravascular coagulation, *DIC score* based on the International Society on Thrombosis and Haemostasis criteria, *p* point, *PT* prothrombin time

**Fig. 5:**
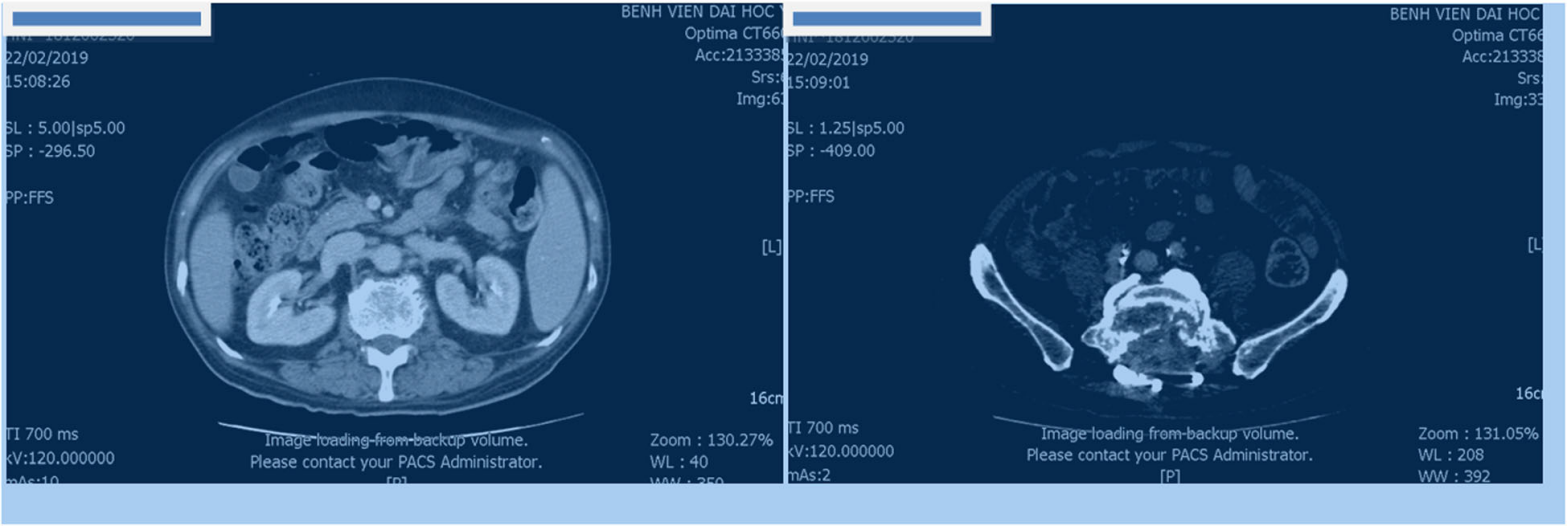
Abdominal computed tomography of patient at second month (February 2019)

**Fig. 6:**
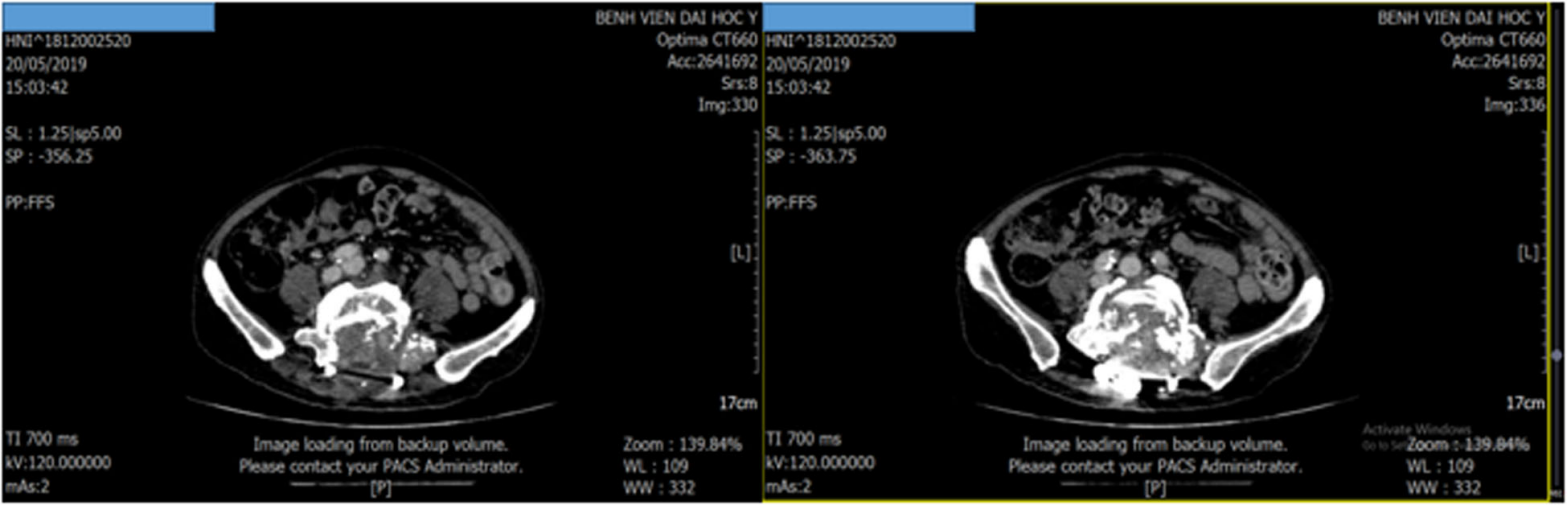
Abdominal computed tomography of patient at fifth month (May 2019)

After the first cycle of systemic chemotherapy, the platelet cell count of our patient recovered to the normal range with 168 × 10^9^/L and his subcutaneous bleeding completely disappeared. He tolerated treatment well without severe adverse events during the period of chemotherapy. After four cycles of paclitaxel plus carboplatin, he was evaluated with an abdominal CT scan and he had achieved a stable disease response at that time and 3 months later, according to the Response Evaluation Criteria in Solid Tumors (RECIST) 1.1 system (Fig. 5, 6). He has been receiving maintenance therapy with sorafenib (400 mg twice daily) up to now. Recently, 11 months subsequent to the diagnosis of DIC, he was still alive and had a good performance status with sorafenib treatment.

## Discussion and conclusion

DIC is a common complication of a number of illnesses including sepsis, trauma, malignancy, and liver disease [5]. A systemic activation of coagulation is an essential capability of DIC, which leads to contribution to fibrin clots and consumption of platelets and coagulation factors. Therefore, the diagnosis of DIC should be based on clinical bleeding, thrombosis, and laboratory information [2]. In a cohort study of 217 consecutive patients in intensive care units (ICUs) who had a clinical suspicion for DIC, 70 patients (32%) were diagnosed as having DIC by the ISTH scoring system. Of these patients, only four cases of DIC (5.7%) had a fibrinogen level under 1 g/L. Thus, platelet count, prolonged prothrombin time, and D-dimer level were mostly encountered in the ISTH DIC score [6]. These findings reflected the contributions to the DIC score of the present patient. In addition, data from the study showed that the proportion of 28-day mortality in patients with DIC and without DIC were 45% and 25%, respectively [6].

In contrast to such acute disease, in which DIC appears as a life-threatening emergency, DIC in cancer might present as a chronic or even subclinical process with only aberrant laboratory results [7]. Some patients have unique thrombocytopenia which is a feature in up to 98% of cases of DIC [2]. The prevalence of DIC occurrence in solid tumors was approximately 7%; in particular, there is a predilection to DIC in elderly patients, male patients, patients with advanced tumors, patients with breast cancer, and with the presence of necrosis in the tumor. Among 7% of cancer-related cases of DIC, RCC was only in 5%, which means that DIC occurrence in RCCs is extremely rare [8]. Hence, only two cases have been reported previously in the literature. According to a study of 1117 patients with solid tumors, cases of overt DIC with advanced tumors had significantly lower survival than cases of non-overt DIC (9 versus 14 months, *p* = 0.005). This finding demonstrated the impact of a serious complication, including DIC, on the outcome of patients with cancer [8].

The basis of the treatment of DIC is to manage the underlying disease [9]. In a cohort of 1117 patients with solid tumors, among 76 cases who were diagnosed as having DIC, one third of these patients achieved response to DIC treatment including replacement therapy (fresh frozen plasma, platelet transfusion, and red blood cell transfusion), heparin, antithrombin III concentrates, and management of the underlying malignancy. In addition, the study indicated that the median survival of the patients with advanced tumors-related DIC was 9 months [8]. Based on our knowledge, we indicated a systemic chemotherapy for our patient to resolve cancer-related DIC prior to a confirmed diagnosis of IHC results.

As mentioned previously, our patient is the first case with successfully managed RCC-related DIC. Of the two reported cases, one case was diagnosed as having subacute DIC in liver metastatic RCC and the other was an autopsy case of pulmonary metastasis of RCC with DIC. In the first reported case, the calculated DIC score by the ISTH was 6 and he died 4 weeks later with only anticoagulation treatment after admission [3]. Currently, RCC is considered a chemotherapy-resistant cancer, in particular in clear cell, which is based on a review of 72 cytotoxic chemotherapy agents in 3502 patients with advanced RCC. The study showed that only 197 cases (6%) had a complete or partial response [10]. Until recently, the mechanisms of chemotherapeutical resistance in RCC remained unclear. Some cytotoxic regimens revealed modest activity, which suggests that chemotherapy might benefit subsets of patients with RCC, such as a rapid tumor growth, to withdraw the development of neoplasm or progress of tumors on cytokines or targeted agents [11]. In the current case, paclitaxel plus carboplatin regimen completely removed DIC; however, he achieved only a stable disease for the evaluation of tumor response after four cycles of chemotherapy. The outcome of our patient might be interpreted as follows: Tissue factor (TF), which is expressed by either the endothelial cells of the vessels or neoplastic cells, binds circulating factor VII(a). This complex enables the activated factors IX and X to trigger systemic activation of coagulation [7]. According to the proportion and number of tumor cells containing TF, a non-overt or overt DIC develops [7]. Interestingly, in the second reported case, the autopsy of a pulmonary metastatic RCC with DIC revealed that most of the vascular endothelial cells were alternated by metastatic carcinoma cells [4]. Thus, it might result in sustaining thrombin generation and consumption of fibrinogen and platelet cells [7]. Alternatively, metastatic dissemination is the last stage in a variety of steps of tumor progression and there is a genetic evolution occurring in the neoplastic cells during their development. Therefore, a primary tumor is probably different from the one contributed by metastatic carcinoma cells in distant organs. Accordingly, the characteristic of chemoresistant incipient clear cell RCC might be alternated in disseminated cells. However, the limitations of this case include the suboptimal regimen of management in RCC and the lack of total laboratory assessment for DIC following the first cycle of chemotherapy. As mentioned previously, the optimal treatment for RCC recently is immunotherapy and molecularly targeted therapy. Due to financial issues and the availability of drugs, this patient could not be treated with the optimal therapy. Also, based on the current data, paclitaxel plus carboplatin is not the optimal regimen in metastatic RCC management, in terms of systemic chemotherapy. The combination of gemcitabine and a fluoropyrimidine (infusion 5-FU or capecitabine) produced the greatest efficacy compared to other chemotherapy regimens, and paclitaxel, which is an anti-microtubule, failed to demonstrate an efficacy in RCC treatment [12]. The rational for the use of paclitaxel-carboplatin regimen in the present case derives from the confirmed diagnosis at the time.

From this patient, cytotoxic chemotherapy still has a crucial role in CCRCC-related DIC management, despite the era of immunotherapy and molecularly targeted therapy in the treatment of metastatic CCRCC and the chemotherapy-resistant feature of these neoplastic cells. An early detection of overt DIC and the indication of systemic chemotherapy without delay are the key to successful cancer-related DIC treatment.

## Data Availability

All data generated or analyzed during this study are included in this published article.
